# Unorthodox Use of Point-of-care Ultrasound to Evaluate Seizures

**DOI:** 10.7759/cureus.3960

**Published:** 2019-01-25

**Authors:** Meryl Abrams, Mark A Magee, Zachary Risler, Resa E Lewiss, Arthur K Au

**Affiliations:** 1 Emergency Medicine, Thomas Jefferson University, Philadelphia, USA

**Keywords:** point of care ultrasound, seizure, posterior shoulder dislocation

## Abstract

It can be difficult to distinguish between syncope and seizure. Some stigmata of seizure include post-ictal period, tongue-biting or incontinence. A less common finding after a seizure is a posterior shoulder dislocation. Posterior shoulder dislocation is commonly missed and may be the only finding after a seizure, thus aiding in diagnosis. In this case report, we discuss the incidence of posterior shoulder dislocations and their utility in differentiating syncope from seizure, as well as the ability to diagnose and evaluate for proper reduction of posterior shoulder dislocations using ultrasound.

## Introduction

Syncope is a frequent chief complaint in the emergency department, accounting for 1%-2% of all emergency department visits [1]. Causes of syncope can be classified into four categories: neurologic, cardiac, orthostatic and neurally mediated [1]. Seizure as a cause of syncope can be difficult to discern as up to 20% of syncopal episodes not due to seizure may have myoclonic jerks [2]. An understanding of the stigmata of epileptic activity may assist in differentiation. The main stigmata include tongue biting, head turning and witnessed posturing [1]. Posterior shoulder dislocation is a less frequently encountered pathology that may suggest a seizure. Comprising only 2%-5% of shoulder dislocations, posterior shoulder dislocations most often occur after seizures, electrocution or trauma [3]. In this case report, we present the case of a 47-year-old man who was brought to the emergency department after a syncopal episode with a posterior shoulder dislocation diagnosed by point-of-care ultrasound (PoCUS).

## Case presentation

A 47-year-old male with a history of hypertension presented to the emergency department after losing consciousness while sitting at his computer. Co-workers witnessed “seizure-like” activity and lowered the patient to the floor. In the emergency department, the patient was amnestic to the episode and complained of right shoulder pain. Review of systems was negative for tongue biting, bowel or bladder incontinence, headache, chest pain, palpitations or shortness of breath. The patient had no past history of seizures. The social history was significant for occasional alcohol consumption without drug use. By history alone, the providers were not sure if the patient had suffered a seizure or other forms of a syncopal episode.

In the emergency department, the patient’s vital signs were blood pressure 156/90 mmHg, heart rate 92 beats per minute, respiratory rate of 17, room air oxygen saturation 97%, and temperature 96.7 degrees Fahrenheit. The physical examination of the patient revealed a male in moderate discomfort from right shoulder pain. The cardiopulmonary examination was unremarkable. There were no focal neurologic deficits. The patient held his right shoulder in adduction with internal rotation. The shoulder was diffusely tender to movement without visible deformity. The patient had intact deltoid muscle sensation, 5/5 muscle strength in the radial, median, and ulnar nerve distributions. He had a 2+ radial pulse. The were no breaks in the skin. Initial work up included a point-of-care of 169 mg/dL; electrocardiogram (EKG) showed normal sinus rhythm with an incomplete right bundle branch block. There was no prior EKG.

**Figure 1:**
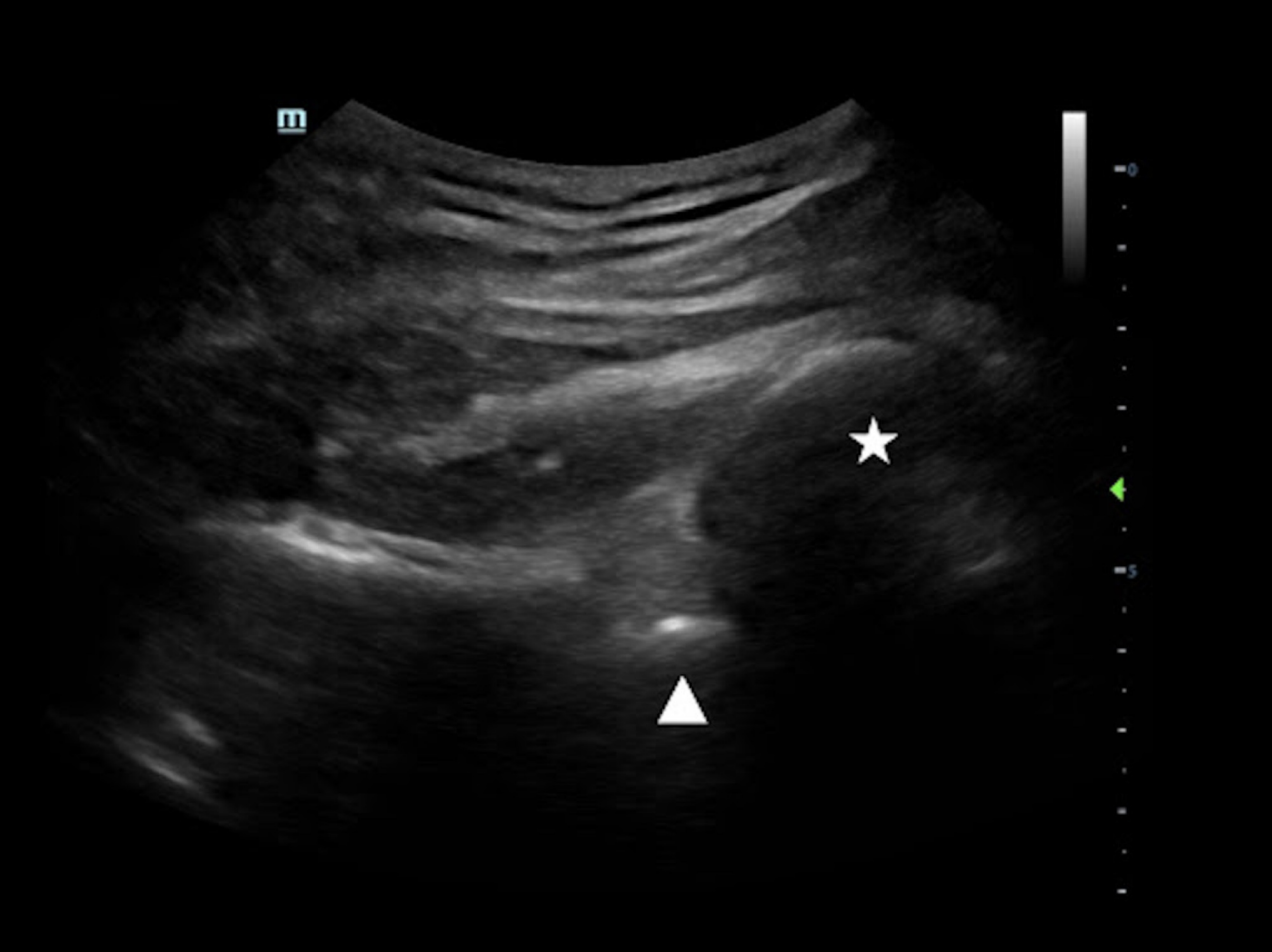
Posterior shoulder dislocation A low-frequency curvilinear transducer is placed on the patient’s posterolateral right shoulder. The humeral head (☆) is posteriorly displaced from the glenoid (△).

**Figure 2:**
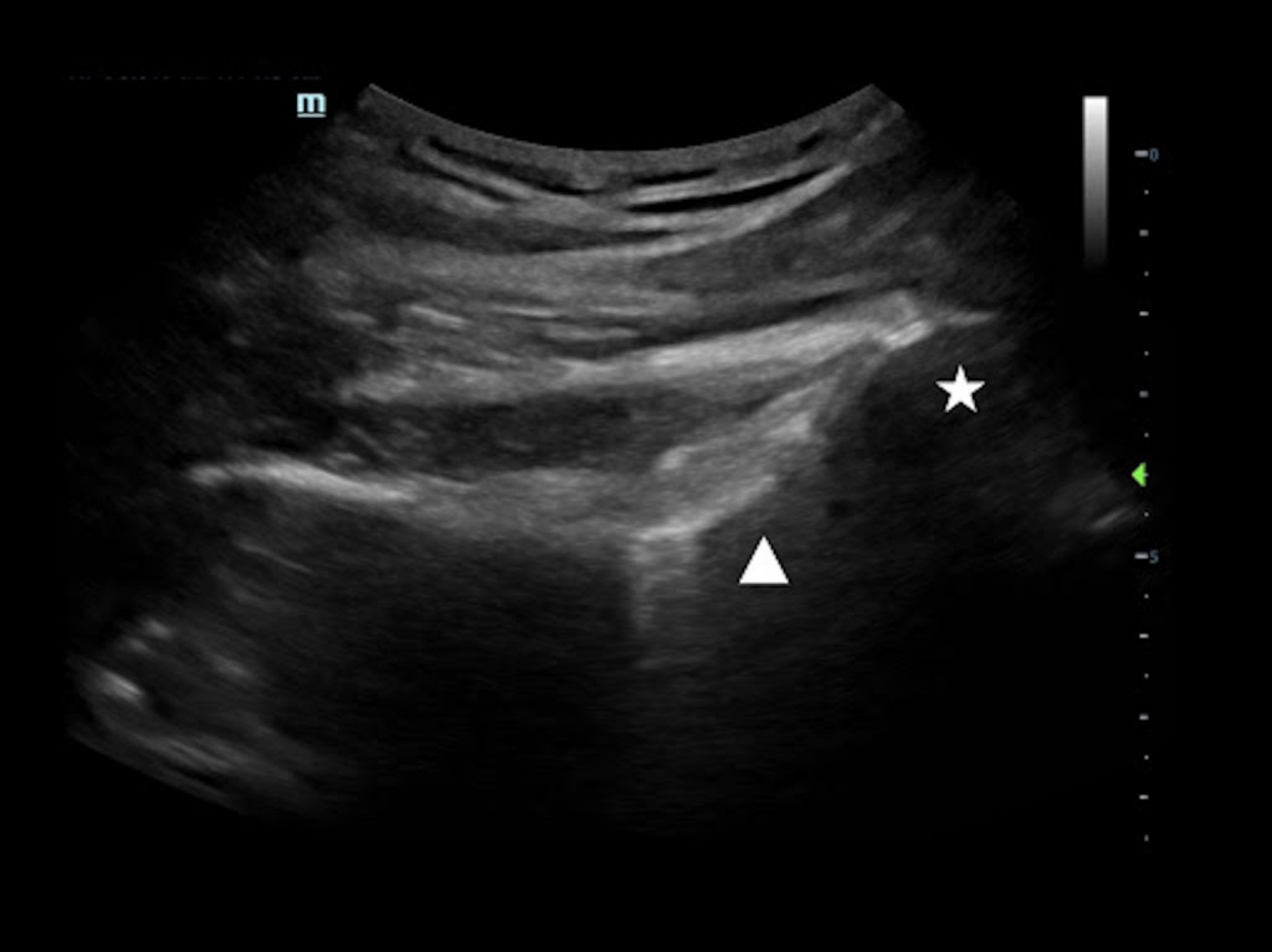
Reduced dislocation of the right shoulder A low-frequency curvilinear transducer is placed on the patient’s posterolateral right shoulder. The humeral head (☆) is aligned the glenoid (△).

A PoCUS of the right shoulder was performed using a low-frequency curvilinear transducer. The humeral head was posteriorly displaced from the glenoid fossa suggesting a posterior shoulder dislocation (Figure 1). Initial radiographs were inconclusive, and the radiologist suggested further views to determine if a dislocation was present. As the dislocation was detected on ultrasound, the patient did not require repeat imaging which both saved time and minimized patient discomfort. The dislocation was reduced using traction counter-traction with the patient under deep sedation with propofol. The successful reduction was then confirmed by both PoCUS and a radiograph (Figure 2). The patient was admitted to the hospital for observation and specialty evaluation by the Orthopedic, Cardiology and Neurology services. He was discharged with the diagnosis of a first-time seizure.

## Discussion

Seizure, a neurologic cause of syncope, can be difficult to distinguish from other causes of syncope, as up to 20% of patients with non-neurologic syncope have myoclonic jerks due to global cerebral hypoperfusion [2]. Gauer described tongue biting, head turning and witnessed posturing as factors that make seizures the most likely cause of syncope, and presyncopal spells, diaphoresis or prolonged standing prior to syncope as factors that make seizures less likely. Common injuries from seizures include tongue lacerations, head trauma, and shoulder dislocations.

Given the relative infrequency of presentation, up to 79% of posterior shoulder dislocation diagnoses are missed or delayed [1]. In a study of 120 dislocations, it was found that most occurred in 20-49 year old men after traumatic events (67%) and seizures (31%) [4]. If a posterior shoulder dislocation is missed, it can lead to chronic dislocation, osteoarthritis or avascular necrosis of humeral head [5]. An X-ray can be used as an initial screening tool for shoulder dislocations; however, findings can be subtle. Ultrasound, however, is up to 100% sensitive and 100% specific for shoulder dislocations [6]. In our case, the diagnosis of a posterior shoulder dislocation using ultrasound helped to support the diagnosis of a first-time seizure.

## Conclusions

The presence of shoulder dislocations can be useful in distinguishing seizures from other causes of syncope. PoCUS is a useful diagnostic tool in the evaluation of both anterior and posterior shoulder dislocations.
